# Supplementary material for: Altered resting-state functional connectivity of the frontal-striatal circuit in elderly with apathy
Source: PLoS One. 2021 Dec 13;16(12):e0261334. doi: 10.1371/journal.pone.0261334 (PMC8668136; doi:10.1371/journal.pone.0261334)
Supplement: Attachment — Submitted filename: Response to Reviewers.docx [file pone.0261334.s002.docx]

Satoshi Ikemoto

Academic Editor

PLOS ONE

U.S. Headquarters

1265 Battery Street, Suite 200

San Francisco, CA 94111 United States

Re: Manuscript ID: PONE-D-21-12168

Dear Prof. Ikemoto,

Thank you very much for your e-mail and review of our manuscript (PONE-D-21-12168) entitled “Altered resting-state functional connectivity of the frontal-striatal circuit in elderly with apathy”. We also appreciate the reviewers taking the time to offer us your comments and insights related to the paper. In the following sections, you will find our responses to each of your points and suggestions.

**Journal requirements:**

**Q.** Please review your reference list to ensure that it is complete and correct. If you have cited papers that have been retracted, please include the rationale for doing so in the manuscript text, or remove these references and replace them with relevant current references. Any changes to the reference list should be mentioned in the rebuttal letter that accompanies your revised manuscript. If you need to cite a retracted article, indicate the article’s retracted status in the References list and also include a citation and full reference for the retraction notice.

**A.** We appreciate the helpful suggestion regarding Journal requirements. We fixed our reference list as follows:

Page 24 Line 399-400

2019; 101(1):165-177.e5. doi:10.1016/j.neuron.2018.11.016

Page 24 Line 409

doi:10.31887/DCNS.2016.18.1/shaber

Page 25 Line 414

doi:10.1523/JNEUROSCI.11-03-00667.1991

Page 25 Line 428

doi:10.1016/sS0006-8993(99)01531-0

**Comments to the Author**

6. Review Comments to the Author

Reviewer #1

**Q.** The authors have addressed most of my concerns well. However, in line 139, the bandpass filter COULD NOT be 0.08-0.09Hz, the typic one should be 0.01-0.09Hz. If this work was done with 0.08-0.09Hz, I do not think these is any signal left for functional connectivity calculation. Please Check.

A. Thank you for your comment. We have confirmed, and the bandpass filter was 0.008, not 0.08. We fixed the bandpass filter as follows:

Page 8 Line 130

band-pass filtering of 0.008–0.09 Hz.

We look forward to hearing from you regarding our submission. We would be glad to respond to any further questions and comments that you may have.

Sincerely,

Chizuko Hamada, M.D.

Department of Neurology, Shimane University

89-1, Enyacho, Izumo, Shimane, Japan, 693-8501

E-mail: okuzihc@med.shimane-u.ac.jp
